# Chinese Expert Consensus on Iodine^125^ Seed Implantation for Recurrent Cervical Cancer in 2021

**DOI:** 10.3389/fonc.2021.700710

**Published:** 2021-11-09

**Authors:** Ping Jiang, Lijuan Zou, Lichun Wei, Guanghui Cheng, Baosheng Sun, Fuquan Zhang, Ruoyu Wang, Tiejun Wang, Ang Qu, Xiangkun Yuan, Bin Qiu, Shuhua Wei, Zi Liu, Yunyan Zhang, Junjie Wang

**Affiliations:** ^1^ Department of Radiation Oncology, Peking University Third Hospital, Beijing, China; ^2^ Department Radiation Oncology, The Second Affiliated Hospital of Dalian Medical University, Dalian, China; ^3^ Department of Radiation Oncology, Xijing Hospital, Fourth Military Medical University, Xi’an, China; ^4^ Department of Radiation Oncology, China-Japan Union Hospital of Jilin University, Changchun, China; ^5^ Department of Radiation Oncology, Jilin Cancer Hospital, Changchun, China; ^6^ Department of Radiation Oncology, Peking Union Medical College Hospital, Beijing, China; ^7^ Department of Radiation Oncology, Affiliated Zhongshan Hospital of Dalian University, Dalian, China; ^8^ Department of Radiation Oncology, The Second Hospital of Jilin University, Changchun, China; ^9^ Department of Radiation Oncology, Cangzhou Hospital of Integrated Traditional Chinese and Western Medicine, Cangzhou, China; ^10^ Department of Radiation Oncology, The First Affiliated Hospital of Xi’an Jiaotong University, Xian, China; ^11^ Department of Radiation Oncology, Harbin Medical University Cancer Hospital, Harbin, China

**Keywords:** radiotherapy, recurrent cervical cancer, brachytherapy, expert consensus, three-dimensional printing template

## Abstract

The treatment modality for recurrent cervical cancer (rCC) is limited, and the prognosis of these patients is poor. Seed implantation could be an important component of rCC management in the context of dose boost or salvage therapy after surgery or radiotherapy, which is characterized by a minimally invasive, high local dose, and rapidly does fall, sparing normal tissue. For patients with good performance status and lateral pelvic wall recurrence with an available puncture path, seed implantation was recommended, as well as for selected central pelvic recurrence and extra-pelvic recurrence. The combination of brachytherapy treatment planning system and CT guidance was needed, and three-dimensional printing templates could greatly improve the accuracy, efficiency, and quality of seed implantation to achieve a potential ablative effect and provide an efficient treatment for rCC. However, the recommendations of seed implantation were mainly based on retrospective articles and lack high-quality evidence, and multicenter prospective randomized studies are needed. In this consensus on iodine^125^ seed implantation for rCC, indication selection, technical process and requirements, dosimetry criteria, radiation protection, combined systemic therapy, and outcomes of seed implantation for rCC are discussed.

## Introduction

Cervical cancer (CC) is the fourth most common female malignancy worldwide. The management for CC includes radical resection (with or without adjuvant radiotherapy) and concurrent chemoradiotherapy (1, 2). The prognosis of patients with complete resection or complete remission after surgery or radiotherapy is excellent, with a 5-year survival rate of about 90% for early-stage and 70% for advanced-stage CC (1). However, recurrent CC (rCC) is still reported in 10–20% of patients with stage IB–IIA and in 40–70% locally advanced disease after initial treatment (3–5). It was found that 31% of the patients relapsed between 18 and 24 months, 58% within 1 year, and 76% within 2 years, and only 6% of these patients with recurrence survived for 3 years (6). Patients with positive pelvic lymph nodes, parametrium invasion, and positive surgical margin were associated with a high risk of postoperative recurrence (7). The percentages of pelvic recurrences fluctuate from 10–74%, depending on different risk factors (3). Recurrences that were distant or detected at multiple sites occurred in 15–61% of patients (4). Most of the patients who developed rCC within 2 years after treatment are associated with a poor prognosis, and most of those patients died of uncontrolled tumors.

The presentation of rCC after initial treatment included local/regional recurrence, distant metastasis, or both, which can be divided into two types: intra-pelvic (IPR) and extra-pelvic recurrence (EPR). The therapy varies from rCC type, mainly including pelvic exenteration, concurrent chemo-radiotherapy (referring to external beam radiotherapy, EBRT), and brachytherapy (BT), while it is still lacking optimal salvage solutions for inoperable and irradiated rCC. Pelvic exenteration is only recommended for select patients yielding a 5-year survival rate of 21%–73%, but the resectable rate is less than 20% and the incidence of postoperative complications is high, with a median survival time of only 7–9 months and a 5-year survival rate of <10% (3, 8).

BT could be an important component of rCC management, with the advantages of highly focused and conformal dose distribution and few damages to the surrounding normal tissues. According to the dose rate, BT could be divided into high-dose-rate BT (HDR-BT) and low-dose-rate BT (LDR-BT); the comparison of HDR-BT and LDR-BT is shown in **Table 1**. At present, HDR-BT with after-loading is mainly used for the primary treatment of CC (9), endometrial cancer (10), breast cancer (11, 12), skin cancer (13), and prostate cancer (14). LDR-BT (mainly referred to as seed implantation) is commonly used for the treatment of various recurrent cancers, such as recurrent head and neck cancers (15, 16), lung cancer (17), rectal cancer (18), and rCC (19, 20). This Chinese expert consensus was focused on iodine^125^ seed implantation for rCC.

**Table 1:** The characteristics of HDR-BT and LDR-BT.

	HDR-BT	LDR-BT
Image guidance	Ultrasound/CT/MRI	CT/ultrasound
Isotope	Iridium^192^	Iodine^125^
Dose rate	High	Low
Implant time	Temporary	Permanent
3D-PCT or 3D-PNCT	+	+
Pre-plan	+	+
Intraoperative plan	+	+/-
Post-plan	–	+
Fraction	2–6	Single
Indication	Limited	Relative no-limitation for location
Protocol	Simple/repeated needle insertion	Simple needle insertion and a quick needle removal

+, necessary; -, not necessary; HDR, high-dose rate; LDR, low-dose rate; BT, brachytherapy; CT, computerized tomography; MRI, magnetic resonance imaging; 3D-PNCT, 3D printing non-co-planar template; 3D-PCT, 3D printing co-planar template.

## Methods

The members of the Brachytherapy and Intelligent Radiotherapy Branch of Chinese Nuclear Society, Chinese Society of Radiation Oncology, Chinese Medical Doctor Association Brachytherapy Professional Committee, and Chinese Northern Radioactive-Seed Brachytherapy Group carried out a literature search. The literature search was using the keywords “brachytherapy” or “seed implantation” and “cervical cancer” and included studies from 1990 to January 2021 as published in PubMed, Embase, ScienceDirect, and Chinese databases. The evidence was then analyzed, and the opinions and suggestions of the experts were formed. The leader organized the experts to write the primary draft, then sent it to all the members for extensive soliciting of opinions, and finally formed a consensus through centralized discussion.

## rCC Diagnosis and Management

### Clinical Diagnosis

The clinical diagnosis of rCC is indicated by symptoms such as weight loss, lower extremity edema, pelvic/lower extremity pain, vaginal bleeding, or routine examination after initial treatment. Image evaluation included ultrasound, CT/MRI scan, and PET-CT in certain circumstances. The final diagnosis relied on pathological confirmation by biopsy under ultrasound or CT guidance. Given the very low proportion of operable patients and recent advances in three-dimensional printing template (3D-PT) and BT technology, individualized and precise treatments are available. IPR may be further subdivided into central pelvic recurrence (CPR) and lateral pelvic wall recurrence (LPR) (21), as shown in **Table 2**. CPR is defined as a recurrent tumor located in the center or midline of the pelvis that may invade anterior (bladder), posterior (rectum), or lateral (vaginal vault) structures, but not the pelvic wall. LPR is defined as invasion of the pelvic wall by the recurrent tumor or adhesion to the pelvic wall or direct invasion of the pelvic wall. EPR includes recurrence in the retroperitoneal lymphatic drainage area, supraclavicular and axillary lymphatic drainage area, mediastinum lymph drainage area, and inguinal lymph node and distance organ metastasis.

**Table 2:** BT and 3D-PT selection according to rCC classification.

Site of recurrence	Subgroup	BT	3D-PT
Intra-pelvic	CPR	HDR/LDR	3D-PNCT
	LPR	LDR/HDR	3D-PNCT
	Inguinal region	LDR	3D-PCT/3D-PNCT
External-pelvic	Retroperitoneal	LDR/HDR	3D-PNCT
	Supraclavicular	LDR	3D-PNCT

rCC, recurrent cervical cancer; BT, brachytherapy; CPR, central pelvic recurrence; LPR, lateral pelvic wall recurrence; 3D-PNCT, 3D printing non-co-planar template; 3D-PCT, 3D printing co-planar template; HDR, high-dose rate; LDR, low-dose rate.

### Management of rCC After Radical Resection

For patients with rCC after radical resection who have not previously undergone radiation therapy, the treatment options include pelvic exenteration and concurrent chemoradiotherapy ± BT. Clinical studies comparing the two treatment modalities are absent, and a multidisciplinary discussion is recommended. Pelvic exenteration is usually indicated for selected patients with CPR (2). The 5-year survival rates for patients with CPR are ranging from 6 to 77%, and patients with CPR seem to have a better prognosis compared to those with LPR (3). However, it is difficult to radically remove LPR by surgery when the tumor is invading the pelvic wall. The effect of neoadjuvant therapy is not defined, and evidence for intraoperative radiotherapy is lacking and not routinely recommended. Concurrent chemoradiotherapy (grade IIIC recommendation) was also recommended for rCC (CPR/LPR) (2). Image-guided radiotherapy may be recommended to ensure accurate irradiation as well as dose-boost of the target area while minimizing the dose to the intestine and other organs at risk (OAR). However, EBRT is still limited by the dose tolerated by normal tissues and the anatomical change after resection, making it usually difficult to meet the curative intent, especially for LPR. Despite the very good results obtained with EBRT and the fact that there has been a trend toward reducing the use of BT, the absence of BT resulted in a high-risk rate of local recurrence (14). Evidence indicated that EBRT seemed not better than BT for dose boost in locally advanced CC, usually with a local lesion boost of 10–20 Gy after 45–50 Gy EBRT (22). Iodine^125^ seed implantation has obvious advantages in therapeutic dose boost. Iodine^125^ seed implantation could be used alone or combined with EBRT for LPR but not encouraged for CPR (only used for selected patients with mass that has a boundary from the vagina).

### Management of rCC After Radiotherapy

The National Comprehensive Cancer Network (NCCN) guidelines provide treatment recommendations for patients with different types of rCC after radiotherapy (2). Pelvic exenteration ± intraoperative radiotherapy could be considered for CPR patients, and BT could be selected for patients with smaller CPR lesions, which is a highly complex procedure and should be performed in high-volume centers (2). As most of the patients who developed rCC after radiotherapy is in the primary locally advanced stage, the usage of pelvic exenteration is limited. Among strictly screened patients, the 5-year survival rate was 30–60% for pelvic exenteration, while the incidence of complications was high, the perioperative mortality rate was 1–10%, and the quality of life decreased significantly (23–25). No preferred treatment is recommended for LPR after EBRT with a 5-year overall survival (OS) rate of <10% and median OS of 7–9 months in general (26). Pelvic exenteration is usually not suitable (27). Given the difficulty in increasing the dose of the clinical target volume (CTV) and dose limitation to OAR for rCC patients after pelvic radiotherapy, re-EBRT and dose boost are difficult due to the dose limitation of OAR (28, 29), while BT is expected to benefit these patients. Seed implantation is characterized by a minimally invasive, high local dose and rapidly does fall. With the advancement of 3D technology, the CTV is accurately determined. 3D-PT is applied in many Chinese BT centers to assist seed implantation with high accuracy (15). It is reasonable to recommend seed implementation only for residual tumors after EBRT.

## Iodine^125^ Seed Implantation for rCC

**Figure 1:**
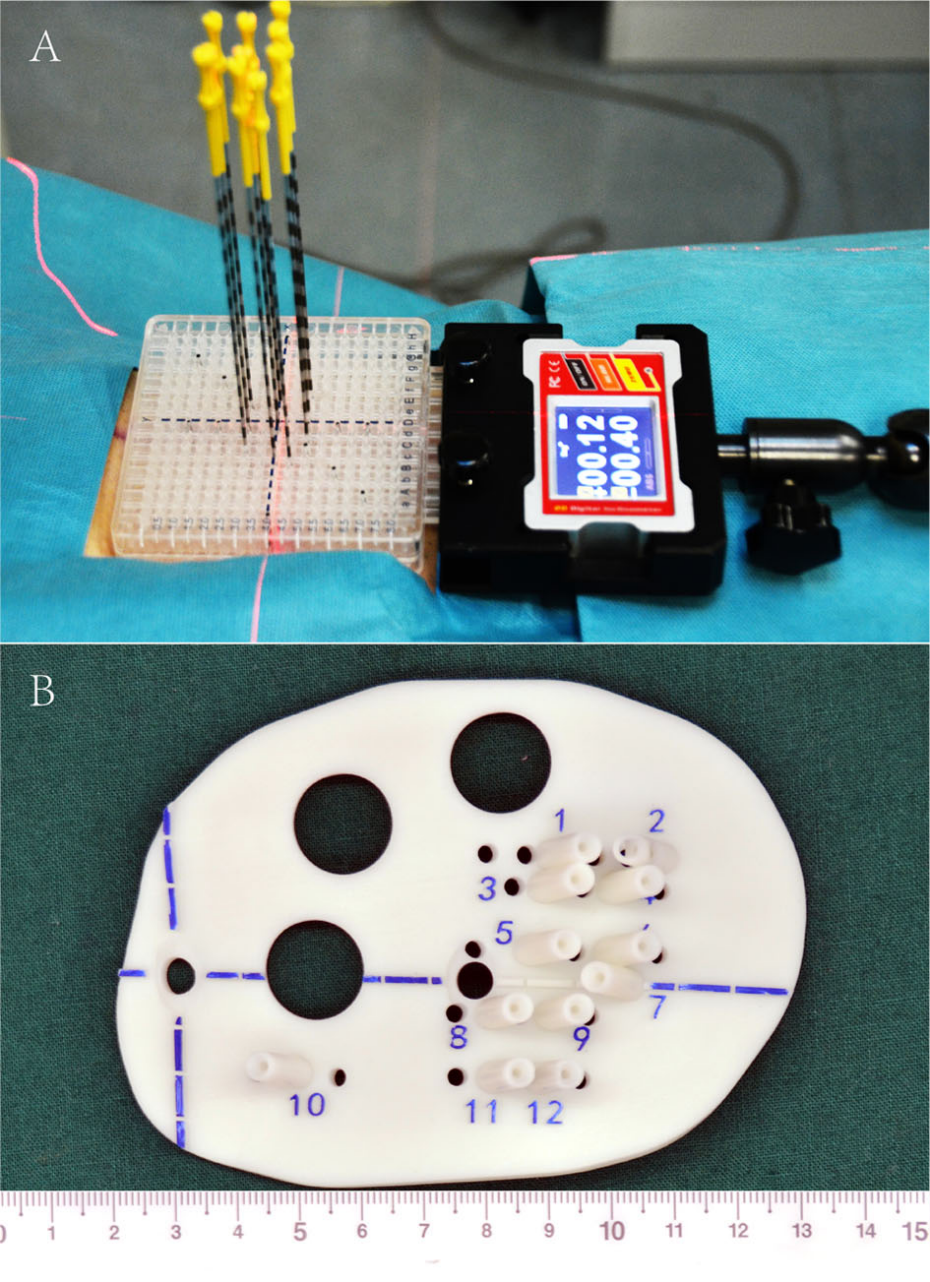
3D printing co-planar template **(A)** and 3D printing non-co-planar template **(B)**.

Iodine^125^ seed implantation has become one of the standard therapies for early prostate cancer, which is comparable to surgery and EBRT, and has been recommended by the NCCN guidelines (30–32). However, iodine^125^ seed implantation is previously rarely reported for rCC. Until 2002, Chinese scholars initialed seed implantation to manage rCC, while several disadvantages shadowed the wide clinical practice: (1) owing to the poor controllability of the needle angle and direction and seed distribution, the learning curve of the clinician was quite long, (2) interference of OAR—radiation distributions to CTV were frequently hard to meet the pre-planning, and (3) frequent repeated CT scan during the implantation increased patient radiation exposure, while ultrasound guidance is two-dimensional imaging with poor accuracy (33–36). Since 2015, individualized 3D-PT was successfully developed to facilitate seed implantation in China (37). 3D-PT was divided into 3D printing co-planar template (3D-PCT) and 3D printing non-co-planar template (3D-PNCT) (**Figure 1**). 3D-PCT applies to the BT with all parallel needle track implants; 3D-PNCT applies to the seed implantation with non-coplanar needle track implants. The technical characteristics of 3D-PT are presented in **Table 3**. The combination of 3D-PT with CT guidance could greatly improve the accuracy, efficiency, and quality of seed implantation to achieve an ablative effect and provide a new and efficient salvage treatment for rCC (15, 37, 38). Until now, the technical process, criteria, and clinical application of 3D-PT-assisted seed implantation for rCC have been discussed in this consensus (20, 39).

**Table 3:** 3D-PCT and 3D-PNCT characteristics.

	3D-PCT	3D-PNCT
Pre-plan	+	+
Intraoperative plan	+/-	+/-
Post-plan	+	+
Needle arrangement	Parallel	Unparallel
Locking needle track	–	+
Individualized needle track	–	+

+, necessary; -, not necessary; 3D-PNCT, 3D printing non-co-planar template; 3D-PCT, 3D printing co-planar template.

### Indication of Seed Implantation for rCC

(1) Age 18–80 years old with Karnofsky Performance Status ≥80;
(2) Pathologically confirmed rCC or residual tumor in patients who are intolerant or refuse surgery [with a diameter of ≤7 cm (35)];
(3) No systemic metastasis or with stable metastasis after systematic treatment (number of lesions ≤3);
(4) Expected survival ≥3 months;
(5) Puncture path is available, and the estimated pre-plan could meet the prescription dose requirements; and
(6) Patients tolerated anesthesia and the seed implantation procedure.

### Contraindications of Seed Implantation for rCC

(1) Severe coagulation disorders with a bleeding tendency (platelets ≤50 × 10^9^/L, prothrombin time >18 s, or prothrombin activity <40%);
(2) Anticoagulant therapy and/or antiplatelet aggregation drugs are taken within 1 week before seed implantation;
(3) Serious complications: severe diabetes, hypertension, heart, lung, renal function insufficiency, infectious period, or immunocompromise;
(4) Patients in a compulsive position, unable to coordinate, and unable to tolerate anesthesia and puncture; and
(5) Tumor invading the rectum or with fistula formation or invading the skin or with skin ulceration.

### Relative Contraindications of Seed Implantation for rCC

(1) Complicated with extensive systemic metastasis or significant local pain;
(2) Allergy to iodine contrast agents; and
(3) Paralysis due to local compression of the spinal cord by the tumor.

### Seed Implantation Prescription Doses for rCC

(1) Prescription dose of seed implantation alone: gross tumor volume (GTV), 110–130 Gy; clinical tumor volume (CTV), 90–110 Gy. The prescription dose of seed implantation combined with/after EBRT: 90–110 Gy for the GTV and 70–90 Gy for the CTV;
(2) Image fusion is recommended for enhanced CT, MRI, and/or PET-CT, with a scanning slice thickness of 5 mm; CTV is formed with a 5- to 6-mm security margin to the GTV; seed activity, 0.4–0.5 mCi.
(3) OAR dose limitation: the dose parameters of OAR during seed implantation for prostate cancer may be used for reference. Bowel D2cc (maximum doses that covered 2-cm^3^ volume) < 100% of prescribed dose, *D*_0.1cc_ (maximum doses that covered 0.1 cm^3^ volume) <200 Gy (35). Rectum D2cc <100% of the prescription doses; D0.1cc <200 Gy (35). Urethral D_10%_ (maximum dose received by 10% of urethral volume) <150% of prescribed dose, D_30%_ (maximum dose received by 30% of urethral volume) <130% of prescribed dose (35). When the recurrent tumor is adjacent to the spine or invades the spinal cord, attention should be paid to prevent nerve injury. At present, the specific dose threshold for nerve injury is still unclear, while the quantitative analyses of normal tissue effects in the clinic dose limitation for the spinal cord is recommended, with the estimated risk of myelopathy being <1 and <10% at 54 and 61 Gy, respectively (40). It is recommended to maintain a 1-cm distance away from the spinal cord during seed implantation and control the seed activity below 0.5 mCi when the spinal cord is nearby.

### Seed Implantation Work Flow for rCC

**Figure 2:**
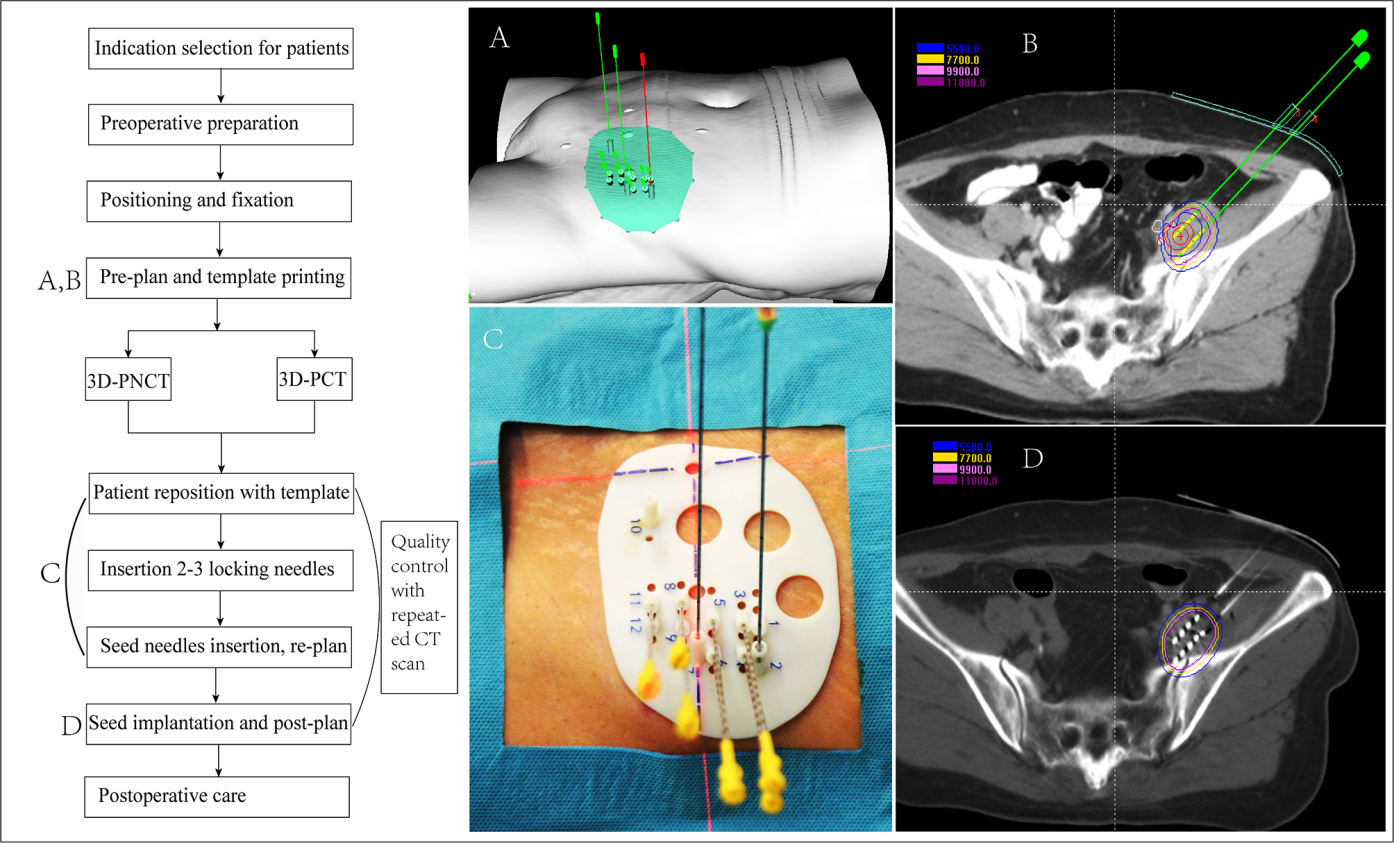
Flow chart of 3D printing template-assisted CT-guided iodine^125^ seed implantation for recurrent cervical cancer. 3D-PCT, 3D printing co-planar template; 3D-PNCT, 3D printing non-co-planar template.

The work flow includes the following steps, each of which requires strict quality control to ensure that the seeds are accurately implanted (39, 41) (the seed implantation requirements for rCC at different locations are recommended in **Table 4** and **Figure 2**):

(1) Preoperative preparation: preoperative evaluation, practicing for the position needed during seed implantation, skin preparation, bowel preparation (EPR/LPR/residue), indwelling catheter (LPR/residue), indwelling vaginal dilator (LPR/residue), *etc.*;
(2) Positioning and fixation: supine or prone position fixation using vacuum pad, enhanced CT scan (slice thickness, 5 mm); setting the tumor center according to the laser rays and mark on the body surface of the patient and vacuum pad;
(3) Preplan: transmit the CT scan images to the brachytherapy treatment planning system (BT-TPS) for atlas of GTV/CTV and definition of OAR; preplan by the physicist according to prescription dose and the dose limitation of OAR given by the clinician;
(4) 3D-PT assistance (optional): the preplan data in the BT-TPS was then transferred into 3D imaging and reverse engineering software for simulation of individualized 3D-PT with a coordinate system, locking needles (used to fix the template)/seed needle pathway (15), and the body surface information of the treatment area; print the 3D-PT according to the simulation using 3D light-cured rapid-forming printer (39);
(5) Patient reposition with the 3D-PT using CT scan to verify the position of locking needles according to the preplan, if the error of the needle tip distance between preoperative CT and intraoperative CT is ≤4 mm, continue to insert other seed needles; if the error is >4 mm, adjust the template position and repeat the above-mentioned steps (39);
(6) Needle insertion: verify the seed needle position by CT scanning after the seed needles are inserted until the error is ≤4 mm; an intraoperative replan may be conducted, if necessary, according to the position of the inserted seed needles: intraoperative real-time CT scan, transmit the image to the BT-TPS, intraoperative needle track verification, real-time planning, and optimization compared with the preplan;
(7) Seed implantation: implant the seed one by one according to the preplan, and the seed needle is removed from the body after seed implantation;
(8) Post-plan: post-operative CT scan: perform CT scan immediately after the seed implantation and transmit the CT images to the BT-TPS for dosimetry evaluation after the seed needle is completely removed (35);
(9) Postoperative care: compress hemostasis and bandaging after removing the insertion needle and template, and send the patient back to the observation room; then, patients with spinal anesthesia should return to the ward with electrocardiography and blood pressure monitoring; and
(10) Follow-up: follow-up is started from the time of seed implantation, and tumor response was first evaluated at 4 weeks and then every 3–6 months thereafter with CT/MRI.

**Table 4:** Iodine^125^ seed implantation for rCC at various sites.

	CPR	LPR	Inguinal region	Retroperitoneal area	Supraclavicular region
Postural fixation	Required	Required	Required	Required	Required
Position type	Supine	Supine	Supine	Prone	Supine
Anesthesia	Epidural	Epidural	Local	Local	Local
Fraction	4–6	Single	Single	Single	Single

rCC, recurrent cervical cancer; BT, brachytherapy; CPR, central pelvic recurrence; LPR, lateral pelvic wall recurrence; HDR, high-dose rate; LDR, low-dose rate.

### Special Notes for Each EPR Location

EPR includes recurrence in lymph nodes and distant organ metastasis. The recurrence rate after concurrent chemoradiotherapy is about 2–12%, and the prognosis is poor (42). For patients with oligometastatic EPR (curative intent) or symptomatic disseminated EPR (palliative intent) for which EBRT was not suitable, seed implantation should be recommended and evaluated. Seed implantation is feasible in patients with EPR after chemoradiotherapy, especially for patients with symptomatic compression by the tumor.

#### Seed Implantation in the Inguinal Region

The patients are in supine position with local anesthesia. A 3D-PNCT/3D-PCT may be used. The seed distribution was 1 cm away from the great vessels and spinal cord, and the activity of the seed should be controlled at 0.4–0.5 mCi. The prescription dose was recommended with 110–160 Gy.

#### Seed Implantation in the Retroperitoneal Region

The patients are in prone position with local anesthesia. 3D-PNCT should be used. The seed distribution was 1 cm away from the great vessels and spinal cord, and the activity of the seed should be controlled at 0.4–0.5 mCi. The prescription dose was recommended with 110–160 Gy.

#### Seed Implantation in the Supraclavicular Region

The patients were in supine position, and local anesthesia was performed. Both head and body fixation techniques were used with 3D-PNCT. The iodine^125^ seed distribution was 1 cm away from the great vessels and brachial plexus, and the activity was controlled at 0.4–0.5 mCi. Pre-operative MRI may be used to atlas OAR such as the brachial plexus.

### Radiological Protection

The half-life of the seed is 59.6 days. The energy is reduced to half of the initial value after 60 days, 10% of the initial value after 6 months, and negligible after 1 year. After seed implantation, the patient should wear the lead vest, collar, or abdominal belt of 0.25-mm-lead equivalent at the implantation site. It is recommended that the discharged patient should maintain a 1-m distance during prolonged contact with their attendant or visitor for 2 months. The patients should not live in a room with children and pregnant women and should not contact or hug children during the first year after seed implantation.

### Combined Systemic Therapy

Platinum-based chemotherapy combined with local therapy is recommended as the first-line treatment for rCC, and the response rate is about 17–30% (43). The response rate was highest for cisplatin in combination with paclitaxel. The survival time of this regimen is reported to be about 13 months, while only 10.0–10.3 months for other combination regimens (44). For those who have previously used cisplatin, carboplatin combined with paclitaxel is recommended; generally, four to six cycles are appropriate. The order of seed implantation and chemotherapy was not well defined, while chemotherapy was usually conducted before (after) seed implantation for patients with relatively diffused (limited) lesions. Targeted therapy was reported in the treatment of CC, and GOG240 III phase clinical study is a landmark advance. Bevacizumab or topotecan combined with paclitaxel improved the survival time from 13.3 to 17 months, with mild adverse reactions ^[84]^. After the local treatment of rCC, first-line chemotherapy combined with bevacizumab is recommended. In case of failure after the first-line chemotherapy, a combination with a second-line chemotherapy regimen is recommended.

### Outcomes of Seed Implantation for rCC

The outcomes of seed implantation for rCC are largely obtained from a single-center retrospective study. The only prospective study (45) by Wang et al. was conducted on 62 patients with rCC after surgery. The patients were randomized into iodine^125^ seed implantation (*n* = 30) and concurrent chemoradiation with EBRT (*n* = 32). The local control rats at 1, 3, 6, and 12 months were higher for iodine^125^ seed implantation than concurrent chemoradiation with EBRT (76.7 *vs*. 65.6%, 80.0 *vs*. 65.5%, 83.3 *vs*. 62.5%, and 86.7 *vs*. 71.9%, respectively). The median OS was 4.34 *vs*. 3.59 years, and the 1-, 2-, 3-, 4-, and 5-year OS were 96.7 *vs*. 81.3%, 93.3 *vs*. 71.9%, 86.7 *vs*. 62.5%, 71.9 *vs*. 56.3%, and 65.6 *vs*. 53.1%, respectively. Han et al. (46) reported 17 patients with rCC who received CT-guided iodine^125^ seed implantation with a median follow-up time of 9.5 months, six patients of whom had a complete response, four patients had a partial response, and seven patients had a progressive disease. The clinical efficacy rate as 58% (10/17). No patient had complications of radiation injury. The rate of 6-month and 1-year survival period was 74.8 and 18.3%, respectively. Compared to patients who responded ineffectively to radioactive seed implantation, patients who responded effectively to radioactive seed implantation had a longer survival period (median 7.2 *vs*. median 10.4), in which the difference was statistically significant (*P* = 0.038). Tong et al. (47) evaluated iodine^125^ seed implantation for 35 patients with rCC after EBRT with a median follow-up of 16 months. The 1-, 3-, 6-, 12-, and 18-month local control rates were 84.5, 74.2, 60.0, 55.5, and 33.3%, respectively. The symptoms significantly improved after implantation. The median local tumor progression-free survival and OS times were 7 months (range, 1–19 months) and 12 months (range, 2–42 months), respectively. The 1- and 2-year OS rates were 65.5 and 43.6%, respectively. Two patients showed grade 3 and 4 toxicity, one patient had a rectovaginal fistula, one patient had incomplete intestinal obstruction, and three cases showed seed migration. No grade 5 event occurred.

#### Iodine^125^ Seed Implantation With 3D-PT

3D-PT-assisted CT-guided iodine^125^ seed implantation is accurate, and it was feasible to obtain favorable dosimetry. Yuan et al. (48) reported 21 patients with postoperative rCC, and they were randomly divided into two groups. One group with 11 patients received 3D-PT, and the other 10 patients received free-hand seed implantation. The D_2cm3_ value of the bladder, rectum, sigmoid colon, and bowel was significantly decreased in the 3D-PT group compared with free-hand seed implantation. 3D-PT guidance has obvious dosimetry advantages in the treatment of rCC and is associated with shorter treatment duration and better repeatability. Qu et al. (39) have investigated the accuracy of needle distribution and dosimetric parameter differences of 3D-PNCT-assisted CT-guided iodine^125^ seed implantation in 38 recurrent gynecological cancer patients with non-central pelvic recurrence between pre-operative plan and post-operative plan. All patients had successfully received 3D-PNCT-assisted seed implantation. No significant differences were shown in D90, D100, V100, V150, V200, and the homogeneity index between pre-operative and post-operative plans. Only a few patients suffered from ≤grade 2 toxicities. Liu et al. (20) reported 103 patients with rCC after EBRT who underwent 3D-PT-assisted CT-guided iodine^125^ seed implantation. The median prescription dose was 120 Gy. The median OS was 17 months, and the 3-year local control rate was 75.1%. Grade 2 adverse events of acute nausea, diarrhea, and pollakiuria occurred in one, two, and one patient, respectively. One patient suffered from grade 3 acute proctitis. Late toxicity was observed in two patients with rectovaginal fistula. No grade 5 toxicity occurred.

#### CPR *vs*. LPR

According to the experience of many BT centers in China over the past 20 years, seed implantation is recommended as salvage therapy for LPR; the seed implantation for LPR seems more common with better outcomes compared with CPR. In the above-mentioned study by Liu et al. (20), only eight lesions were CPR, 75 lesions were LPR, and 28 lesions were EPR. Studies directly comparing seed implantation for CPR and LPR were lacking, while the outcome differences between these two groups of populations were observed from a sub-group analysis. Qu et al. (19) reported 36 patients with rCC (15 CPR and 21 LPR) who received CT-guided iodine^125^ seed implantation after EBRT. With a median follow-up of 11.4 months, the 1- and 2-year local progression-free survival (LPFS) rate was 34.9 and 20%, respectively. The 1- and 2-year OS rate was 52.0 and 19.6%, respectively. The multivariate analysis indicated that recurrence sites (CPR or LPR) were the independent factors for both LPFS and OS (hazard ratio = 0.294 and 0.358, respectively). Qu et al. (28) reported 39 patients with rCC who were treated by image-guided seed implantation. The OS of patients with CPR and LPR was 6 and 12 months, respectively, and the 1-year progression-free survival rate was 26.7 and 41.6%, respectively, suggesting that the prognosis of seed implantation for LPR was superior to that of CPR, with a low incidence of side effects.

#### Seed Implantation for Specific EPR

The studies on seed implantation of EPR were mainly reported for lymphatic metastasis from CC, but along with other cancers, the complications were acceptable, while the survival outcomes may be biased by the mixed analysis with other cancers. 3D-PT-assisted CT-guided iodine^125^ seed implantation is also feasible. Jiang et al. (38) reported 15 patients with 17 retroperitoneal recurrent carcinomas after EBRT (26.7% from CC). All patients received CT-guided iodine^125^ seed implantation assisted by 3D-PNCT. No ≥grade 3 adverse reactions were observed. The preliminary clinical study showed that CT-guided iodine^125^ seed implantation assisted by 3D-PNCT was a safe, accurate, and feasible strategy for recurrent carcinomas located in the retroperitoneal regions. Chen et al. (49) reported 32 patients with retroperitoneal recurrent lymphatic metastasis carcinomas after EBRT who successfully underwent 3D-PNCT-assisted seed implantation. A total of 81.3% of the patients achieved pain relief, and 71.9% were improved. The overall response rate and the local control rate were 85.3 and 94.1%, respectively. The local control rates reached 66.2 and 43.2% in 1 and 2 years, respectively, with a median local control time of 15.8 months. The 1- and 2-year OS rates were 74.1 and 28.1%, respectively, with a median OS of 17.6 months. Except for two patients developing grade 1 retroperitoneal hematomas, no other severe adverse events were observed. Guo (50) reported 14 patients with supraclavicular metastatic tumor (15 lesions) who received 3D-PT-assisted CT-guided iodine^125^ seed implantation. The difference in D90, V100, V150, V200 (percentage of GTV receiving 100 or 150 or 200% of the prescription dose, respectively), matched peripheral dose, and conformal index between pre- and post-operation was not statistically significant (*P* > 0.05). The external volume index (defined as the ratio of the non-target volume received dose ≥ prescribed dose to target volume) of pre-operation was significantly higher than that of post-operation (55.8 *vs*. 33.4, *P* = 0.02). It was concluded that personalized 3D-PT-assisted CT-guided iodine^125^ seed implantation for supraclavicular metastatic tumor is accurate and feasible. Further efficacy study focusing on EPR from CC only is warranted.

## Author Contributions

PJ wrote the manuscript draft. JW, YZ, LZ, and BQ reviewed and revised it. All authors contributed to the article and approved the submitted version.

## Funding

This work was supported by the National Key Research and Development Program of 413 China (grant no. 2019YFB1311300) and the National Natural Science Foundation of China (grant no. 82073335) to JW.

## Conflict of Interest

The authors declare that the research was conducted in the absence of any commercial or financial relationships that could be construed as a potential conflict of interest.

## Publisher’s Note

All claims expressed in this article are solely those of the authors and do not necessarily represent those of their affiliated organizations, or those of the publisher, the editors and the reviewers. Any product that may be evaluated in this article, or claim that may be made by its manufacturer, is not guaranteed or endorsed by the publisher.
